# Evaluating the Utility of Carbon Isotope Discrimination for Wheat Breeding in the Pacific Northwest

**DOI:** 10.34133/2019/4528719

**Published:** 2019-08-29

**Authors:** Liam S. Dixon, Jayfred V. Godoy, Arron H. Carter

**Affiliations:** Washington State University, Washington, USA

## Abstract

Many wheat (*Triticum aestivum* L.) production regions are threatened annually by drought stress. Carbon isotope discrimination (*Δ*) has been identified as a potentially useful trait in breeding for improved drought tolerance in certain environments. Broad use of *Δ* as a selection criterion is limited, however, mainly due to an inconsistent relationship observed between grain yield and *Δ* and, to a lesser extent, because of the high resource demand associated with phenotyping. The efficiency of selection may be improved by the identification and verification of molecular markers for use in marker-assisted selection (MAS), and a reliable relationship to grain yield may be established based on a location's total amount and distribution of precipitation over the growing season. Given the environmental variability in precipitation dynamics, it is necessary to evaluate this relationship in target breeding environments. In this study, grain *Δ* was collected on a panel of 480 advanced soft white winter wheat varieties grown in five Pacific Northwest environments. A genome-wide association study approach was used to evaluate the amenability of grain *Δ* to MAS. The genetic architecture of grain *Δ* was determined to be characterized by multiple, small effect marker-trait associations with limited repeatability across environments, suggesting that MAS will be ineffective at improving *Δ* selection efficiency. Further, the relationship between grain yield and *Δ* ranged from neutral (*r* = ‐0.01) to moderately positive (*r* = 0.44) in the target environments. Such moderate correlations, coupled with variability in this relationship, indicate that direct selection for *Δ* may not be beneficial.

## 1. Introduction

Drought stress is the single greatest threat to wheat (*Triticum aestivum* L.) productivity worldwide, impacting more than half of all growing regions [1, 2]. As one of Washington State's most widely cultivated crops, wheat and the associated industries are critical to rural economies and to the State economy as a whole [3]. Like much of the world, drought represents one area of significant vulnerability to Washington's wheat growers [4]. In 2015, drought stress resulted in an estimated 22 percent reduction in wheat yield, representing a total economic loss of $212.4 million [5]. Though an extreme case, this was not an isolated event. Washington's major wheat-producing area is among the most drought-prone regions in the country [6].

Plant breeding efforts to develop more tolerant varieties of wheat may be able to mitigate the impacts of drought. Breeding for drought tolerance is complicated, however, largely due to the quantitative nature and significant genotype-by-environment interactions associated with drought tolerance. Past efforts targeting drought tolerance directly based on yield components have experienced limited success [7]. An alternative strategy, involving selection for less complex physiological traits that are associated with yield in dry environments (leaf rolling, stay green, canopy temperature depression, osmotic adjustment, water soluble carbohydrates, deep roots, and glaucousness), has been suggested as a more effective approach [8]. Many of these traits are less influenced by the environment and can therefore contribute to greater yield stability [9].

Carbon isotope discrimination (*Δ*) has been identified as an indirect selection criterion for improving grain yield in certain environments [10, 11]. In the early 1980s, researchers observed that the molar concentration ratio of ^13^C relative to ^12^C in plant tissue is often lower than the same ratio of atmospheric carbon. This finding indicates that plants discriminate in favor of ^12^C during the conversion of CO_2_ into biomass. As a standard for evaluating plant isotopic composition, Δ was proposed by Farquhar and Richards [12] and is defined as the deviation from unity of atmospheric ^13^C/^12^C over plant ^13^C/^12^C.

Wide genetic variation in *Δ* exists among and between plant species. This variation may be largely explained by genotypic differences in stomatal conductance and photosynthetic capacity. Early research found *Δ* to share a positive and linear relationship with the ratio of internal leaf CO_2_ concentration (*C*_i_) and the atmospheric CO_2_ concentration (*C*_a_) [13]. This ratio is principally impacted by stomatal conductance and photosynthetic capacity [14]. A high *Δ* genotype may therefore be assumed to express high stomatal conductance, low photosynthetic capacity, or both, whereas the opposite may be assumed for a low *Δ* genotype. The relationship to *C*_i_/*C*_a_ makes it possible to draw inferences regarding fundamental plant physiology based on measurements of *Δ*. Further, *Δ* can be highly heritable and relatively easy to manipulate in breeding programs [15, 16].

Despite these positive characteristics, common use of *Δ* as a selection criterion has been limited. This is largely due to the variable relationship observed between *Δ* and grain yield across environments and, to a lesser extent, because of the resources necessary to phenotype. Selection efficiency may be improved by the identification and verification of molecular markers associated with *Δ* for use in marker-assisted selection (MAS). A genome-wide association study (GWAS) can be considered one of the first steps in identifying potential candidate markers for MAS [17]. By way of statistical analysis, a GWAS detects DNA markers that share an association to the phenotype [18, 19]. The results provide researchers with a better understanding of the trait's genetic architecture, including the number of marker-trait associations (MTAs) involved in trait expression as well as the relative contribution of each genetic region to the observed phenotype. For effective use in MAS, the identified DNA markers should explain a high proportion—typically between 40 and 60%—of the trait's phenotypic variation and demonstrate repeatability across environments [11, 20, 21]. Although *Δ* is influenced by both stomatal conductance and photosynthetic capacity, traits that are polygenic themselves, there is evidence for moderate effect markers, explaining 30% of the phenotypic variance [22], as well as marker repeatability [23]. These findings suggest that *Δ* may be amenable to MAS, warranting further examination.

Reasons for the inconsistent grain yield and *Δ* relationship are not well understood, though differences in environmental conditions, principally the distribution and amount of precipitation, may be large factors. Most field studies conducted under moderate water limitations report either a positive or neutral relationship between grain yield and *Δ* [24–28]. Only infrequently are negative correlations observed, occurring most often in very dry environments where crops experience little precipitation during the growing season and rely on stored soil moisture as a water source [10, 15, 29]. For effective use as a selection criterion, the relationship to grain yield in a particular environment ought to be consistent from year-to-year, either positive or negative, and of reasonably high correlation (typically *r* > 0.5 or *r* < ‐0.5). The complexity between *Δ* and grain yield reported in the literature, along with the potential to improve yield under drought stress, makes it appropriate to study this relationship in target environments where breeding lines are tested.

The relationship between *Δ* and grain yield has not been evaluated for the primary wheat-producing region of Washington State. Precipitation across this region follows a low (<300 mm) to high (450-600 mm) gradient moving east from the foothills of the Cascade Mountains to the Washington-Idaho border [30]. The winter wheat breeding program at Washington State University targets two main environments—low and high precipitation. The objective of this study was to explore the relationships between *Δ* and grain yield and, more broadly, to evaluate the utility of *Δ* as a selection criterion to target breeding environments in Washington State. To this end, practical elements of selection are considered beyond the trait's relationship to yield, including trait heritability, genetic variation, and ease of selection.

## 2. Materials and Methods

### 2.1. Plant Material

This study collected data on a panel of 480 advanced soft white winter wheat varieties from U.S. Pacific Northwest breeding programs (Oregon State University, University of Idaho, Washington State University, USDA-ARS, and private breeding companies). All varieties included in the panel were from the soft white market class, of which 63% were lax-headed, and the remaining 37% were club head type. From the 480 lines in the original panel, a total of 459 lines were ultimately included in the association analysis. The 21 excluded lines were either identified as a separate phenotypic class, had poor DNA quality, or were missing marker covariate information. Previous work on this panel established the genotype data for each variety [31]. The panel was genotyped using an Illumina Infinium iSelect 90K single nucleotide polymorphism (SNP) chip at the USDA-ARS Biosciences Research Laboratory in Fargo, ND, USA.

### 2.2. Experimental Design

**Figure 1:**
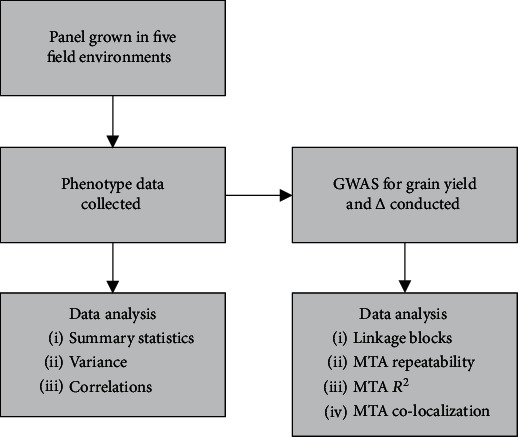
Flowchart of major elements in experimental design, including field growth, phenotyping, GWAS, and data analysis.

A panel of 480 advanced soft white winter wheat varieties was grown in an unreplicated augmented block design in five field environments. The panel was phenotyped for plant height (cm), heading date (Julian), grain yield (t ha^−1^), and *Δ*. Location-specific monthly precipitation throughout the growing season was obtained from databases of nearby weather stations. A GWAS was conducted on the panel for grain yield and *Δ*. MTAs determined to be significant were analyzed for physical linkage, repeatability across environments, percentage of phenotypic variation explained (*R*^2^), and colocalization with significant grain yield MTAs. Summary statistics, as well as inter- and intraenvironment phenotypic variance, for each trait was calculated. Pearson correlations between *Δ* and grain yield, plant height, and heading data were also determined (Figure 1).

### 2.3. Field Conditions

The panel was grown in an augmented block design with unreplicated 2.8 m^2^ plots at the Spillman Agronomy Farm near Pullman, WA (46°7′N; -117°1′W), during 2015, 2016, and 2017. In 2017, the panel was also grown in an augmented block design with unreplicated 6.1 m^2^ plots near Lind, WA (46°8′N; -118°6′W), and 8.7 m^2^ plots near Pendleton, OR (45°7′N; -118°6′W). In each location-year, 20% of the plots were planted to the check cultivar “Madsen” (PI 511679) [32]. Trials were planted in Pullman on October 8, 2014, October 8, 2015, and November 3, 2016; in Lind on September 7, 2016; and in Pendleton on October 4, 2016. Trials were harvested in Pullman on July 23, 2015, August 5, 2016, and August 10, 2017; in Lind on July 20, 2017; and in Pendleton on July 25, 2017. The mean annual precipitation for these locations (2010-2017) is 230 mm at Lind, 435 mm at Pullman, and 235 mm at Pendleton. The mean annual temperature (2010-2017) for Pullman is 8.8°C. In Lind and Pendleton, the mean annual temperature (2010-2017) is 10.2°C and 11.8°C, respectively. Precipitation and temperature data for each location-year were collected from weather stations near the field sites. AgWeatherNet sites (https://weather.wsu.edu/) were used to access precipitation data for all Pullman environments, as well as Lind. An AgriMet weather station (https://www.usbr.gov/pn/agrimet/wxdata.html) entitled “Echo” was used to access precipitation data for the Pendleton field site. This station is approximately 38 miles from the Pendleton field location, whereas the two AgWeatherNet stations are at the same location as the Pullman and Lind field sites.

The amount of precipitation that each location-year received during the growing season ranged from low in Pendleton (265 mm) and Lind (294 mm) to high in Pullman 2016 (463 mm). Moderate levels of precipitation were experienced in Pullman 2015 (384 mm) and Pullman 2017 (377 mm) (Table S1). Both Lind and Pendleton had fairly consistent precipitation throughout the vegetative stages. Pullman 2016 and Pullman 2015 experienced a general pattern of declining precipitation after the seedling stage. Pullman 2017 received high early precipitation and high precipitation in March, with relatively little precipitation during December and January. All environments tended to experience a decrease in precipitation from spring into summer. Pullman 2015 received the least postanthesis precipitation (10 mm) followed by Lind (14 mm), Pullman 2017 (21 mm), and Pullman 2016 (32 mm). Although heading date was not collected for Pendleton 2017, the final two months of the growing cycle received 19.3 mm of precipitation.

### 2.4. Phenotypic Data

Plot heading date (Julian) was determined as the number of days from January 1 to 50% of fully exposed heads. Heading date was recorded in all trials except in Pendleton 2017. Plant height (cm) was measured from the base of the plant to the top of fully emerged heads, excluding the awns. Grain yield (t ha^−1^) was determined from the grain weight per plot obtained from a ZÜRN 150 plot combine harvester (ZÜRN Harvesting, Germany). Grain samples (20 g) were collected from harvested seed for each variety and milled into flour using an UDY cyclone sample mill (UDY Corporation, CO). Flour samples weighing between 3.00 and 5.00 mg were packed into sterile tin capsules. Grain carbon isotope percentages were measured using an isotopic mass spectrometer at the Stable Isotope Core Laboratory at Washington State University. Carbon isotope composition (*δ*^13^C) was calculated by comparing the ratio of ^13^C to ^12^C for each sample (*R*_s_) against the same ratio of a Vienna Pee Dee Belemnite (VPDB) standard (*R*_VPDB_) by the following formula: *δ*^13^C(permil) = [(*R*_s_/*R*_VPDB_) − 1] × 1,000. The *Δ* value of the sample was then calculated as Δ = (*δ*^13^C_a_ − *δ*^13^C_p_)/(1 + *δ*^13^C_p_), where *δ*^13^C_a_ and *δ*^13^C_p_ are the carbon isotope compositions of the atmosphere and plant samples, respectively [13]. The *δ*^13^C_a_ value was assumed to be −8.0 per mil [33]. Carbon isotope composition values are unitless, though they are often expressed in terms of “per mil,” indicating that the original value was multiplied by 10^3^. Doing so is a matter of convenience, allowing values to appear as whole numbers.

### 2.5. Statistical Analysis

The phenotypic data were assessed for influential outliers (greater than three standard deviations). Three suspicious data points were identified in Pullman 2015, six in Pullman 2016, four in Pullman 2017, two in Lind 2017, and four in Pendleton 2017. These outliers were excluded from any further statistical analyses. Pearson correlations and summary statistics (mean, range, and standard deviation) were calculated for phenotypic traits using the R statistical software (R Core Team, 2016). Given the potential to confound the relationship between grain yield and *Δ*, a separate correlation analysis was conducted between grain yield and *Δ* excluding those genotypes with heading date greater than one standard deviation and less than negative one standard deviation from the environment-specific mean. Variation between environments and between genotypes and checks within environments was calculated using SAS version 9.4 (SAS Institute Inc., Cary, NC, USA). ANOVA coupled with post hoc Tukey HSD and linear contrast tests calculated in R statistical software were used to identify any significant differences between environments for phenotypic traits.

Broad-sense heritability for each trait was determined based on the formula *H*^2^ = *V*_G_/*V*_P_, where *V*_G_ is the genetic variance and *V*_P_ is the phenotypic variance [34]. The *V*_P_ for each trait was calculated by dividing the sum of squared residuals of an equal means model by the number of observations. The environmental variance, *V*_E_, was calculated as the sum of the squared residuals for each genotype across environments divided by the total observations. The relationship *V*_P_ = *V*_G_ + *V*_E_ allowed for an estimate of *V*_G_ and subsequently an estimate of *H*^2^ for each trait.

Best linear unbiased predictions (BLUPs) were calculated for *Δ* and grain yield using the lme4 package in R. The mixed linear model considered genotype and environment as random effects. Due to the potential to confound *Δ* results, both heading date and plant height were added to the *Δ* mixed linear model as covariates. The proportion of phenotypic variance explained by significant SNPs (*R*^2^) identified in each location-year and BLUP analysis was calculated in JMP v. 8.1 by fitting genotype and phenotype data on a stepwise regression model and computing the difference in variance explained between a model including all significant SNPs and a model excluding the marker under consideration.

### 2.6. Linkage Disequilibrium (LD)

Linked SNPs determined to be significantly associated with the phenotype were evaluated for physical linkage. The intersection of the locally weighted polynomial regression- (LOESS-) based curve of the LD parameter *r*^2^ and the 95^th^ percentile of the unlinked *r*^2^ distribution was considered to be the critical threshold beyond which LD was due to physical linkage [35]. Previous research on the panel used for this study determined that SNPs with an *r*^2^ value greater than 0.18 and within 4 cM could be considered to be on the same linkage block [36]. The pairwise LD parameter *r*^2^ was computed in TASSEL version 5.2.25 [37].

### 2.7. Association Analysis

Association analysis for grain yield and *Δ* was performed on both the location-year phenotypic data and the BLUP data using 15,229 high-quality SNP markers. Those markers with more than 20% missing data or with a minor allele frequency (MAF) less than 0.05 were excluded from the association analysis. An assessment of the panel's population structure was previously conducted [36]. In the grain yield and *Δ* association analyses, model fit was verified by evaluating quantile-quantile plots (Figures S1 and S2). In the *Δ* association analysis, principal components 1 and 2 were included in the final model to account for the population structure. If heading date or plant height was significantly (*p* < 0.001) correlated with *Δ* in a particular environment, the covariate was added to the final model for that environment. Heading date was added as a covariate to Pullman 2015 and Lind 2017. Plant height was added as a covariate to all environments excluding Lind 2017. The BLUP analysis included both plant height and heading date as covariates. For the grain yield association analysis, model fit did not improve with the addition of principal components, so a model with no covariates was ultimately selected. The purpose of this analysis was to identify significant grain yield MTAs that colocalize with significant *Δ* MTAs, making a basic model for grain yield association analysis appropriate. That said, in addition to a colocalization analysis with no covariates in the grain yield model, the same analysis was conducted using a grain yield model including principal components 1 and 2. No notable differences in the regions or number of MTAs found to colocalize were identified between the two analyses.

Fixed and random Circulating Probability Unification (FarmCPU), implemented in GAPIT2 [38], was used for the marker-trait association analysis. Associations were determined to be significant by the Bonferroni correction method [39] with *α* = 0.05—equivalent to a marker-wise threshold *P* value of 3 × 10^‐6^ (0.05/15,229 SNPs). As the Bonferroni correction is overly conservative, assuming that each association test is independent of all other tests [40], the colocalization assessment considered SNPs significant with a marker-wise threshold *P* value of 1 × 10^‐5^.

## 3. Results

### 3.1. Phenotypic Variance of Measured Traits and Heritability

A wide variation was observed for all measured traits in each environment (Table 1). The mean grain yield varied from 3.0 t ha^−1^ in the Lind 2017 trial to 7.8 t ha^−1^ in the Pullman 2017 trial. The variation for grain yield was significant between the five environments (*P* < 0.0001), as well as between genotypes within each environment (*P* < 0.0001). A post hoc analysis revealed that all five environments were significantly different for grain yield. Mean *Δ* was lowest in the Lind 2017 trial (16.5) and highest in the Pendleton 2017 trial (18.2). The range of *Δ* values within environments was roughly 2.0 per mil for all environments excluding Pullman 2015, which had a range of 3.1 per mil. The variation in mean *Δ* values between environments was significant (*P* < 0.0001), as was the variation in *Δ* values between genotypes within each environment (*P* < 0.0001). A post hoc analysis revealed that Pullman 2017 and Pendleton 2017 were the only environments without a significant difference for *Δ* values (*P* = 0.321). In addition to grain yield and *Δ*, a significant variation for plant height and heading date was observed both between environments (*P* < 0.0001) and between genotypes within each environment (*P* < 0.0001). Considering the Madsen check cultivar only, there were no significant differences for *Δ* (*P* = 0.7409), grain yield (*P* = 0.6632), plant height (*P* = 0.5257), or heading date (*P* = 0.9984) within each environment, providing evidence for limited spatial field variation. Based on this evidence, no data transformations or adjustments were conducted. Broad-sense heritability for *Δ* considering all environments together was determined to be 0.15, whereas broad-sense heritability for grain yield was 0.06 (Table S2). Broad-sense heritability of *Δ* calculated for the Pullman location-years separately increased to 0.27.

### 3.2. Correlation Analysis of Agronomic Traits and *Δ*

**Figure 2:**
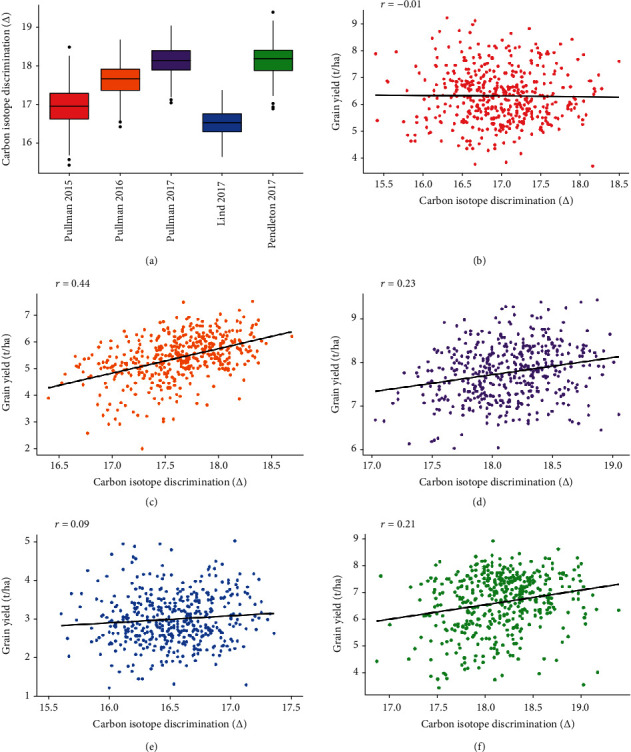
Boxplot of carbon isotope discrimination (*Δ*) across five environments (a) and environment-specific scatterplots of grain yield (t ha^−1^) and *Δ* (b–f) collected from a diverse panel of winter wheat grown in five Pacific Northwest environments—(b) Pullman 2015, (c) Pullman 2016, (d) Pullman 2017, (e) Lind 2017, and (f) Pendleton 2017.

Grain yield and *Δ* were positively correlated (*P* < 0.05) in all environments, excluding Pullman 2015, where no correlation was observed (Table 2; Figure 2). For those environments with a positive relationship, Pearson phenotypic correlations ranged from 0.09 in Lind 2017 to 0.44 in Pullman 2016. Plant height and heading date were both significantly (*P* < 0.05) correlated with *Δ* in all environments for which the data was collected. The Pearson correlations between plant height and *Δ* ranged from -0.13 in the Lind 2017 trial to -0.49 in the Pullman 2017 trial. The range of Pearson phenotypic correlations between heading date and *Δ* varied from -0.09 in Pullman 2016 to -0.25 in Pullman 2015. In a separate analysis, in which early and late heading genotypes were excluded, the correlation between grain yield and *Δ* for each environment remained similar to the original correlation analysis (Table 2).

### 3.3. Model Selection for Association Analysis

Model fit for the grain yield and *Δ* association analyses was verified with quantile-quantile plots (Figures S1 and S2). In the *Δ* association analysis, principal components 1 and 2 were included in the final model to account for the population structure. If heading date or plant height was significantly (*P* < 0.001) correlated with *Δ* in a particular environment, the covariate was added to the final model for that environment. Heading date was added as a covariate to Pullman 2015 and Lind 2017. Plant height was added as a covariate to all environments excluding Lind 2017. The BLUP analysis included both plant height and heading date as covariates. For the grain yield association analysis, model fit did not improve with the addition of principal components, so a model with no covariates was ultimately selected. The purpose of this analysis was to identify significant grain yield MTAs that colocalize with significant *Δ* MTAs, making a basic model for grain yield association analysis appropriate. That said, in addition to a colocalization analysis with no covariates in the grain yield model, the same analysis was conducted using a grain yield model including principal components 1 and 2. No notable differences in the regions or number of MTAs found to colocalize were identified between the two analyses.

### 3.4. Analysis of Significant MTAs

A total of 25 MTAs were significant (*P* < 3 × 10^‐6^) for grain *Δ* across 13 chromosomes (Table 3). The number of significant MTAs varied by environment ranging from two identified in Pendleton 2017 to six in Lind 2017. There were five significant MTAs in the Pullman 2015 environment. In Pullman 2016 and Pendleton 2017 and in the BLUP analysis, four MTAs were found to be significant. The majority of these MTAs were detected on the B genome (14), followed by the A genome (8) and the D genome (3). The most informative chromosomes were 4B, with four MTAs, and 5B, with six MTAs. None of the identified MTAs explained greater than 10% of the observed phenotypic variation (*R*^2^), and only four explained greater than 5%.

Four MTAs and three linkage blocks were found to be significant (*P* < 0.00001) in more than one environment (Tables S3 and S4). However, none of these regions were significant in more than two environments. Two repeated regions were identified on chromosome 4B. On chromosome 5B, two repeated MTAs and one repeated region were identified. Significant (*P* < 0.00001) MTAs for *Δ* identified in a particular environment were assessed for colocalization with significant (*P* < 0.00001) MTAs for grain yield identified in that same environment (Table S5). One linkage block on chromosome 6B was found to be significant (*P* < 0.00001) for both *Δ* and grain yield in the Lind 2017 environment. SNP IWB56415 (60.8 cM) was significant for *Δ* in Lind 2017, and SNP IWB29847 was significant for grain yield (64.7 cM). The pairwise LD *r*^2^ value between these SNPs was 0.24. In the BLUP analysis, a second linkage block common to *Δ* and grain yield was identified on chromosome 7B (*P* < 0.00001). SNP IWB5954 (171.11 cM) was found to associate with *Δ*, and SNP IWB12006 (171.11 cM) was significant for grain yield. The pairwise LD *r*^2^ value between these SNPs was 0.85.

## 4. Discussion

### 4.1. Correlation Analysis of Agronomic Traits and *Δ*

This study found *Δ* to be positively correlated with grain yield in four of the tested environments and neutral in one of the tested environments (Figure 2). These results are consistent with positive correlations reported in previous studies in which grain *Δ* was measured from wheat grown under moderate drought conditions [24–28]. Various hypotheses explain this association between grain yield and *Δ*. High *Δ* genotypes may achieve high productivity as a result of deep roots that allow access to water [11]. Alternatively, stem reserve carbohydrates, fixed under well-watered conditions, can elevate the overall grain *Δ* signature once translocated from stem tissue to grain tissue [28]. High *Δ* grain tissue can also result from stomatal insensitivity to drought, which may be a beneficial trait to a moderate terminal drought environment [11]. Finally, high yield from high *Δ* genotypes may be the result of an early heading date, allowing the genotype to avoid terminal drought conditions.

Variation in heading date may have been partly responsible for the positive correlations observed in this study. Heading date was significantly associated with *Δ* in every tested environment. The broadest range in heading date among genotypes spanned 18 days in Pullman 2017, whereas the narrowest range was 11 days in Pullman 2015. The water availability between early and late heading genotypes was likely significant and undoubtedly impacted grain *Δ* measurements. That said, after removing those genotypes with heading date greater than one standard deviation and less than negative one standard deviation from the mean in each environment, very little difference in the Pearson correlation was observed between grain yield and *Δ* (Table 2), suggesting that the impact of heading date on the relationship between *Δ* and yield may be a minor factor in this study.

A number of studies report a repeated and high correlation value between grain yield and *Δ*, leading researchers to propose grain *Δ* as a predictive selection criterion for wheat grain yield [10, 42–45]. In the present study, such use of grain *Δ* cannot be recommended, as the observed correlation was generally moderate. Further, the relationship between grain yield and *Δ* was inconsistent within the two target breeding environments—high (450-600 mm) and low (<300 mm) precipitation. The high precipitation environment of Pullman saw a neutral relationship in 2015 (*r* = ‐0.01; 384 mm precipitation) and a moderately positive relationship in 2017 (*r* = 0.23; 377 mm precipitation), and in Pullman 2016, the relationship was the most positive of all environments (*r* = 0.44; 463 mm precipitation). As for the low precipitation environments, Lind 2017 had a slightly positive correlation (*r* = 0.09; 294 mm precipitation) while the relationship increased in Pendleton 2017 (*r* = 0.21; 265 mm precipitation). This variation within target breeding environments, combined with the generally moderate correlation observed between grain yield and *Δ*, makes it difficult to recommend direct selection of grain *Δ* in either high or low precipitation environments.

### 4.2. Analysis of Significant MTAs

The genetic architecture of *Δ* is complex. This appears to be so for *Δ* measured on unstressed plant tissue, as well as tissue exposed to drought stress. In the case of unstressed tissue, the *Δ* signature may reflect genetic factors of both stomatal conductance and photosynthetic capacity. These traits are themselves polygenic, and so, a complex genetic architecture may be expected when combined in *Δ* analysis [11]. Fittingly, a total of 54 QTL have been reported for *Δ* across a number of mapping studies [8]. In a biparental mapping study of *Δ* from unstressed leaf tissue, Rebetzke et al. [23] report *Δ* QTL on chromosomes 1A, 1D, 2A, 2B, 2D, 3B, 4A, 4B, 4D, 5A, 5B, 6B, 6D, 7A, and 7B. The identified QTL were of small genetic effect, each explaining less than 10% of the additive genetic variance [23]. In the only other known association analysis where *Δ* is considered, Mora et al. [46] report significant associations of grain *Δ* on chromosomes 1A, 3A, 4A, 4B, 4D, 5A, 5B, 5D, 6B, and 7D, with maximum explained phenotypic variation of 9.7% [46].

In the event that grain fill coincides with a moderate to severe drought, the genetic architecture of grain *Δ* is likely to be even more complex. Along with genetic contributions of photosynthetic capacity and stomatal conductance, grain *Δ* may also be impacted by genetic factors indirectly associated with stomatal conductance, including flowering time, root characteristics, and reliance on stem reserve carbohydrates for grain fill [11]. The grain *Δ* signature depends mainly on the stomatal conductance and photosynthetic capacity occurring at the time of carbon assimilation [11]. Exposure to drought tends to reduce stomatal conductance, driving *Δ* values down [26]. Those genotypes able to maintain open stomata by accessing water deep in the soil profile likely register higher *Δ* values. Similarly, stem reserves laid down prior to the drought onset may also register as high *Δ*, provided fixation occurred during a period of relatively high stomatal conductance. Upon translocation to the grain, these high *Δ* carbohydrates will elevate the *Δ* signature of the kernel tissue [10]. Alternatively, high grain *Δ* could result from early flowering [11]. As plants mature into the later months of summer, it is typical of Mediterranean climates for precipitation and humidity to decline and for temperature to increase. These changes elevate the threat of drought and the likelihood of stomatal closure. A genotype with an earlier heading date may simply be high for *Δ* by avoiding a period of heightened drought potential.

The relationship that *Δ* has with *C*_i_/*C*_a_ makes it possible to approximate the severity of terminal drought in a particular environment. In a well-watered environment, stomatal conductance can be expected to be high, resulting in increased *C*_i_. A higher instantaneous *C*_i_ elevates the overall *Δ* signature. Conversely, as soil moisture becomes scarce, stomatal conductance tends to decrease, lowering *C*_i_ and, ultimately, the *Δ* signature [11]. As such, the average *Δ* of all genotypes from a particular environment can provide an indication as to the severity of terminal drought. Unstressed plants typically register *Δ* values between 19 and 22 per mil [11]. Being that the highest average *Δ* in this study was 18.2 per mil, there is evidence to suggest that each environment experienced a certain degree of terminal drought. On the other end of the spectrum, a severely stressed plant may register *Δ* values of 12 to 14 per mil [11]. The lowest average *Δ* signature observed in the current study was 16.5 per mil, indicating high, but not severe, stress. In terms of *C*_i_/*C*_a_ values, the least severe terminal drought environment experienced average internal carbon concentrations of roughly 0.58, whereas the most severe saw average values of around 0.51 [11]. A difference in average instantaneous *C*_i_/*C*_a_ of 7% over the period of grain carbon assimilation has the potential to result in large cumulative differences in biomass development. On the continuum from severe to moderate terminal drought stress, the environments included in this study are ordered as follows: Lind 2017, Pullman 2015, Pullman 2016, Pullman 2017, and Pendleton 2017.

Given that each environment experienced some level of terminal drought, it is likely that the *Δ* phenotype data was impacted by stomatal conductance, photosynthetic capacity, flowering time, root depth, and the translocation of stem reserves to varying degrees by genotype. The significant SNPs identified in this study may, therefore, also be associated with these contributing traits, excluding flowering time, as the addition of heading date as a covariate in the GWAS model helped to eliminate any associations due to genotypic differences in flowering time. Given the polygenic nature of each contributing trait [8, 47, 48], the dispersed genetic architecture for grain *Δ* revealed in this study is sensible. In a biparental mapping study involving grain *Δ* analysis on genotypes exposed to controlled terminal drought conditions, Peleg et al. [22] detected QTL on chromosomes 1A, 4A, 5A, 5B, 6B, and 7B. Both of the regions on 5B explained 27% of the observed variance. In the current study, chromosome 5B also appeared to be influential to *Δ* expression, with the greatest number of significant MTAs, as well as three repeated regions across environments. Notably, however, the phenotypic variance explained by these MTAs was much lower, the highest being just 7.5%.

While regions on chromosome 5B and others were found to repeat across environments, in general, the associated regions appeared to be largely environment specific. Such genotype-by-environment interactions also appeared to be evident in the grain *Δ* association analysis performed by Mora et al. [46], reporting all SNPs to be particular to each environment. Conversely, Rebetzke et al. [23] described several repeated QTL both across populations and between environments. However, none of the stable chromosomes (2B, 6D, 7A, and 7B) indicated were found to repeat across environments in this study.

Incidentally, phenotypic differences driven by genotype-specific reactions to the drought stress may also help to explain the relatively low heritability for *Δ* observed in this study. Broad-sense heritability for grain *Δ* is often relatively high, ranging from 0.40 [26] to 0.83 [49]. Intermediate values have been reported [26, 50]. Wu et al. [51] estimated a low broad-sense heritability for grain *Δ* of 0.23. The low heritability reported in this study is likely the result of high environmental variance, as evidenced by the significant differences in average *Δ* between environments, and the associated genotype-by-environment interactions. To improve heritability, it may be advisable to measure *Δ* on less stressed leaf tissue prior to the onset of terminal drought, as proposed by Condon et al. [11].

## 5. Conclusion

Grain *Δ* was determined to be a largely ineffective selection criterion for crop improvement in Washington target breeding environments. The low heritability combined with an inconsistent and generally weak correlation with grain yield makes selection for grain *Δ* unfavorable. The variation in environments greatly impacted the ability to use *Δ* as a selection tool. In areas with more consistent environmental factors, selection may be useful; however, in production regions with high variation between locations and years, *Δ* does not prove to be a valuable selection criterion. By measuring *Δ* on less stressed leaf tissue, it is possible that the genotype-by-environment interactions associated with grain *Δ* may diminish, ultimately improving heritability. The genetic architecture of grain *Δ* was found to be characterized by multiple, small effect, and largely environment-specific MTAs, further complicating the use of grain *Δ* as a selection criterion. These results suggest that grain *Δ* from wheat exposed to terminal drought is not amenable to MAS, as no single marker resulted in a large phenotypic response.

## Figures and Tables

**Table 1:** Data summary for grain yield (GY, t ha^−1^), *Δ* (per mil), plant height (HT, cm), and heading date (HD, Julian) collected on a panel of 480 advanced soft white winter wheat lines adapted to the Pacific Northwest.

Trait	Pullman 2015			Pullman 2016			Pullman 2017			Lind 2017			Pendleton 2017		
	Mean	Range	SD^a^	Mean	Range	SD	Mean	Range	SD	Mean	Range	SD	Mean	Range	SD
GY	6.3	3.7-9.2	0.98	5.4	2.0-7.6	0.83	7.8	6.0-9.4	0.63	3.0	1.2-5.0	0.65	6.6	3.4-8.9	1.09
*Δ*	17.0	15.4-18.5	0.53	17.6	16.4-18.7	0.40	18.1	17.0-19.1	0.37	16.5	15.6-17.4	0.34	18.2	16.9-19.4	0.41
HT	89.9	71.1-127.0	7.64	89.0	68.6-121.9	7.41	96.5	76.2-129.5	7.59	80.2	53.3-101.6	7.41	109.5	78.7-142.2	8.41
HD	151.2	144.0-155.0	2.13	156.5	147.0-160.0	2.12	164.8	154.0-172.0	3.23	150.2	142.0-155.0	2.02	N/A	N/A	N/A

^a^Standard deviation.

**Table 2:** Pearson phenotypic correlations between *Δ* and grain yield (GY), grain yield having removed genotypes with early and late heading dates (altGY), plant height (HT), and heading date (HD) for a winter wheat panel grown in five Pacific Northwest environments.

Environment	GY	altGY	HT	HD
2015 Pullman	-0.01	0.02	-0.15^‡^	-0.25^‡^
2016 Pullman	0.44^‡^	0.47^‡^	-0.24^‡^	-0.09^∗^
2017 Pullman	0.23^‡^	0.19^‡^	-0.49^‡^	-0.13^†^
2017 Lind	0.09^∗^	0.14^†^	-0.13^†^	-0.17^‡^
2017 Pendleton	0.21^‡^	N/A	-0.23^‡^	N/A

^∗^ *P* < 0.05, ^†^*P* < 0.01, and ^‡^*P* < 0.001.

**Table 3:** Markers associated with grain carbon isotope discrimination (*Δ*) phenotype collected on a panel of 480 advanced soft white winter wheat lines adapted to the Pacific Northwest and grown in five environments.

Chromosome^a^	Environment^b^	SNP ID^c^	SNP name^d^	Pos (cM)^e^	*P* value^f^	Alleles^g^	MAF^h^	*α* ^i^	*R* ^2^ ^j^
1A	Pullman 2017	IWB57005	RAC875_c34888_65	34.70	1.11*E*-10	**T**/C	0.49	−	0.30
1A	BLUP	IWB10679	BS00073585_51	78.33	6.77*E*-09	T/**G**	0.45	−	3.20
1A	Lind 2017	IWB10679	BS00073585_51	78.33	2.25*E*-07	T/**G**	0.45	−	1.68
2A	Pullman 2017	IWB42693	Kukri_c22686_422	25.97	2.77*E*-12	T/**G**	0.22	−	4.70
2A	Pullman 2015	IWB24017.1	Excalibur_c23681_317	109.20	4.29*E*-09	A/**C**	0.44	−	3.50
2B	Pullman 2016	IWB315	BobWhite_c12144_216	19.72	4.69*E*-07	A/**G**	0.05	−	2.80
2D	Lind 2017	IWB79221	wsnp_Ex_rep_c66522_64795143	61.65	2.33*E*-09	**A**/G	0.30	+	4.18
3B	Pullman 2016	IWB48816	Kukri_rep_c102888_154	62.57	2.69*E*-09	A/**G**	0.49	−	8.50
3D	Pullman 2015	IWB34605	IAAV2729	74.42	1.26*E*-07	T/**C**	0.32	+	7.60
4B	Lind 2017	IWB49195	Kukri_rep_c106598_51	38.30	1.29*E*-08	A/**G**	0.23	+	3.98
4B	Lind 2017	IWB75382	wsnp_BE422566B_Ta_1_2	60.39	1.88*E*-07	**T**/C	0.06	+	2.08
4B	Pullman 2017	IWB65668	TA003248-0911	68.45	4.44*E*-08	**A**/C	0.42	+	0.50
4B	Pullman 2016	IWB36159	IACX6482	78.96	5.70*E*-08	**T**/A	0.43	−	2.90
4D	BLUP	IWB45192	Kukri_c4210_240	93.06	2.54*E*-07	**T**/G	0.31	−	4.32
5B	Pendleton 2017	IWB78507	wsnp_Ex_c58091_59534826	25.18	3.90*E*-07	**G**/A	0.42	−	7.50
5B	Pullman 2015	IWB76070	wsnp_CAP8_c1210_739429	29.11	4.01*E*-07	T/**C**	0.16	−	2.60
5B	Lind 2017	IWB8684	BS00056147_51	48.46	2.93*E*-10	T/**C**	0.42	−	6.68
5B	BLUP	IWB27067	Excalibur_c49597_579	68.36	5.19*E*-07	**T**/C	0.05	−	4.94
5B	Pendleton 2017	IWB27067	Excalibur_c49597_579	68.36	3.30*E*-08	**T**/C	0.05	−	3.20
5B	Pullman 2015	IWB57214	RAC875_c36779_148	89.55	3.38*E*-07	**T**/C	0.14	+	1.60
6A	Pullman 2016	IWB75682	wsnp_BF483091A_Ta_2_5	135.86	3.42*E*-10	**T**/C	0.13	−	7.70
6B	BLUP	IWB12455	BS00109999_51	87.33	5.54*E*-10	T/**C**	0.07	+	4.79
7A	Pullman 2017	IWB22879	Excalibur_c16933_788	59.91	4.79*E*-07	**T**/C	0.06	+	1.40
7A	Lind 2017	IWB12655	CAP11_c1048_99	126.80	1.28*E*-07	**T**/C	0.29	+	3.18
7B	Pullman 2015	IWB52272	Ra_c56305_654	71.33	1.92*E*-08	A/**G**	0.12	+	3.90

^a,c,d,e^Chromosome, SNP ID, SNP name, and chromosome position are all based on the wheat 90K consensus map [41]; ^b^environment from which marker was identified as significantly associated with *Δ*; ^f^nominal *P* values; ^g^alleles for specific SNP markers; the underlined base represents the minor allele; the bold base represents the favorable allele if low *Δ* is desired; ^h^minor allele frequency (MAF). Frequency of minor allele in the panel; ^i^alpha (*α*) denotes the SNP effect on the phenotype; ^j^percent phenotypic variation explained by the SNP.

## Supplementary Materials

Supplementary 1 Table S1: monthly and total precipitation (mm) over the wheat growing season for five environments including Pullman (2015, 2016, and 2017), Lind 2017, and Pendleton 2017.

Supplementary 2 Table S2: variance components for calculation of broad-sense heritability (*H*^2^) including total phenotypic variance (*V*_P_), environmental variance (*V*_E_), and genetic variance (*V*_G_) for grain yield (GY, t ha^−1^), *Δ*, plant height (HT, cm), and heading date (HD, Julian) of a winter wheat panel grown in five Pacific Northwest environments.

Supplementary 3 Table S3: markers associated with carbon isotope discrimination (*Δ*) repeated in more than one environment.

Supplementary 4 Table S4: determination of linkage blocks by pairwise evaluation of markers associated with carbon isotope discrimination (*Δ*) that are equal to or less than 4 cM apart and are in significant linkage disequilibrium (*r*^2^ > 0.18).

Supplementary 5 Table S5: markers associated with grain yield from a panel of 480 advanced soft white winter wheat lines adapted to the Pacific Northwest and grown in five environments.

Supplementary 6 Figure S1: quantile-quantile plots for association analysis of carbon isotope discrimination (*Δ*) including principle components 1 and 2, and covariates for plant height and heading date provided a significant (*P* < 0.001) Pearson correlation with *Δ* evaluated for each location-year including (A) Pullman 2015, (B) Pullman 2016, (C) Pullman 2017, (D) Lind 2017, (E) Pendleton 2017, and (F) best linear unbiased prediction (BLUP).

Supplementary 7 Figure S2: quantile-quantile plots for association analysis of grain yield including no covariates for each location-year including (A) Pullman 2015, (B) Pullman 2016, (C) Pullman 2017, (D) Lind 2017, (E) Pendleton 2017, and (F) best linear unbiased prediction (BLUP).
