# Supplementary material for: Evaluating the Utility of Carbon Isotope Discrimination for Wheat Breeding in the Pacific Northwest
Source: Plant Phenomics. 2019 Aug 29;2019:4528719. doi: 10.34133/2019/4528719 (PMC7706333; doi:10.34133/2019/4528719)
Supplement: Supplementary 1 — Table S1: monthly and total precipitation (mm) over the wheat growing season for five environments including Pullman (2015, 2016, and 2017), Lind 2017, and Pendleton 2017. [file 4528719.f1.docx]

**Table S1** Monthly and total precipitation (mm) over the wheat growing season for five environments including Pullman (2015, 2016, and 2017), Lind 2017, and Pendleton 2017

| Year | Location | Sep. | Oct. | Nov. | Dec. | Jan. | Feb. | March | April | May | June | July | Total |
| --- | --- | --- | --- | --- | --- | --- | --- | --- | --- | --- | --- | --- | --- |
| 2015 | Pullman |  | 25.9 | 65.5 | 77.5 | 47.2 | 54.1 | 59.9 | 10.2 | 34.3 | 4.8 | 4.8 | 384.3 |
| 2016 | Pullman |  | 38.9 | 49.8 | 106.2 | 68.3 | 31.5 | 82.3 | 36.8 | 16.8 | 18.5 | 13.7 | 462.8 |
| 2017 | Pullman |  |  | 39.4 | 29.7 | 23.1 | 65.8 | 121.2 | 36.6 | 39.6 | 20.8 | 0.5 | 376.7 |
| 2017 | Lind | 8.9 | 86.9 | 25.1 | 6.6 | 19.8 | 52.3 | 45.5 | 25.1 | 10.4 | 13.5 | 0.0 | 294.1 |
| 2017 | Pendleton | | 44.2 | 12.4 | 32.8 | 39.6 | 45.0 | 36.1 | 25.9 | 9.4 | 17.8 | 1.5 | 264.7 |
