# Supplementary material for: Evaluating the Utility of Carbon Isotope Discrimination for Wheat Breeding in the Pacific Northwest
Source: Plant Phenomics. 2019 Aug 29;2019:4528719. doi: 10.34133/2019/4528719 (PMC7706333; doi:10.34133/2019/4528719)
Supplement: Supplementary 2 — Table S2: variance components for calculation of broad-sense heritability (H2) including total phenotypic variance (VP), environmental variance (VE), and genetic variance (VG) for grain yield (GY, t ha−1), Δ, plant height (HT, cm), and heading date (HD, Julian) of a winter wheat panel grown in five Pacific Northwest environments. [file 4528719.f2.docx]

**Table S2** Variance components for calculation of broad-sense heritability (H^2^) including total phenotypic variance (V_P_), environmental variance (V_E_), and genetic variance (V_G_) for grain yield (GY t ha^-1^), ∆, plant height (HT, cm), and heading date (HD, Julian) of a winter wheat panel grown in five Pacific Northwest environments

| Trait | V_P_ | V_E_ | V_G_ | H^2^ |
| --- | --- | --- | --- | --- |
| GY | 3.30 | 3.12 | 0.18 | 0.06 |
| ∆ | 0.59 | 0.51 | 0.09 | 0.15 |
| HT | 153.78 | 114.34 | 39.44 | 0.26 |
| HD | 39.37 | 34.79 | 4.58 | 0.12 |
