# Supplementary material for: Evaluating the Utility of Carbon Isotope Discrimination for Wheat Breeding in the Pacific Northwest
Source: Plant Phenomics. 2019 Aug 29;2019:4528719. doi: 10.34133/2019/4528719 (PMC7706333; doi:10.34133/2019/4528719)
Supplement: Supplementary 3 — Table S3: markers associated with carbon isotope discrimination (Δ) repeated in more than one environment. [file 4528719.f3.docx]

**Table S3** Markers associated with carbon isotope discrimination (∆) repeated in more than one environment

| Chromosome^a^ | SNP ID^b^ | SNP name^c^ | Pos (cM)^d^ | Environment 1^e^ | Environment 2^e^ |
| --- | --- | --- | --- | --- | --- |
| 1A | IWB10679 | BS00073585_51 | 78.33 | Lind 2017 | BLUP |
| 5B | IWB27067 | Excalibur_c49597_579 | 68.36 | Pendleton 2017 | BLUP |
| 5B | IWB57214 | RAC875_c36779_148 | 89.55 | Pullman 2015 | Pullman 2017 |
| 6A | IWB79072 | wsnp_Ex_rep_c102807_87894833 | 85.07 | Pullman 2015 | Pullman 2017 |

^a,b,c,d^ Chromosome, SNP ID, SNP name, and chromosome position are based on the wheat 90K consensus map [41]

^e^ Environments from which marker was identified as significantly associated with ∆
