# Supplementary material for: Evaluating the Utility of Carbon Isotope Discrimination for Wheat Breeding in the Pacific Northwest
Source: Plant Phenomics. 2019 Aug 29;2019:4528719. doi: 10.34133/2019/4528719 (PMC7706333; doi:10.34133/2019/4528719)
Supplement: Supplementary 4 — Table S4: determination of linkage blocks by pairwise evaluation of markers associated with carbon isotope discrimination (Δ) that are equal to or less than 4 cM apart and are in significant linkage disequilibrium (r2 > 0.18). [file 4528719.f4.docx]

**Table S4** Determination of linkage blocks by pairwise evaluation of markers associated with carbon isotope discrimination (∆) that are equal to or less than 4 cM apart and are in significant linkage disequilibrium (r^2^ > 0.18)

| Chr^a^ | Region (cM)^b^ | SNP 1 ID^c^ | SNP 1 Name^d^ | SNP 1 Environment^e^ | SNP 2 ID^f^ | SNP 2 Name^g^ | SNP 2 Environment^h^ | Distance Apart (cM) | r^2^ Value^i^ |
| --- | --- | --- | --- | --- | --- | --- | --- | --- | --- |
| 4B | 78.96 | IWB36159 | IACX6482 | Pullman 2016 | IWB23338 | Excalibur_c19547_128 | BLUP | 0 | 1.00 |
| 4B | 68.45-72.53 | IWB65668 | TA003248-0911 | Pullman 2017 | IWB81016 | wsnp_Ra_c22026_31453420 | Pendleton 2017 | 4.08 | 0.61 |
| 5B | 68.36-71.64 | IWB30911 | Excalibur_rep_c68003_954 | Pullman 2016 | IWB11517 | BS00087678_51 | Pullman 2017 | 3.28 | 0.31 |

^a,b,c,d,f,g^ Chr (Chromosome), Region (cM), SNP 1 ID, SNP 1 Name, SNP 2 ID, and SNP 2 Name are based on the wheat 90K consensus map [41]

^i^ Pairwise linkage disequilibrium parameter, r^2^
