# Supplementary material for: Evaluating the Utility of Carbon Isotope Discrimination for Wheat Breeding in the Pacific Northwest
Source: Plant Phenomics. 2019 Aug 29;2019:4528719. doi: 10.34133/2019/4528719 (PMC7706333; doi:10.34133/2019/4528719)
Supplement: Supplementary 5 — Table S5: markers associated with grain yield from a panel of 480 advanced soft white winter wheat lines adapted to the Pacific Northwest and grown in five environments. [file 4528719.f5.docx]

**Table S5** Markers associated with grain yield from a panel of 480 advanced soft white winter wheat lines adapted to the Pacific Northwest and grown in five environments

| Chromosome^a^ | Environment^b^ | SNP ID^c^ | SNP name^d^ | Pos (cM)^e^ | P-value^f^ | Alleles^g^ | MAF^h^ | α^i^ | R^2j^ |
| --- | --- | --- | --- | --- | --- | --- | --- | --- | --- |
| 1B | Pendleton 2017 | IWB38627 | Ku_c17846_363 | 24.55 | 1.15E-06 | T/**C** | 0.46 | - | 8.35 |
| 1B | Pullman 2016 | IWB39151 | Ku_c31363_2165 | 31.04 | 1.06E-06 | T/**G** | 0.25 | + | 1.85 |
| 2A | Pendleton 2017 | IWB1987 | BobWhite_c26296_83 | 25.97 | 1.19E-07 | **T**/C | 0.27 | + | 3.01 |
| 2A | Pullman 2015 | IWB67648 | Tdurum_contig12556_183 | 151.29 | 2.04E-09 | A/**G** | 0.17 | - | 3.07 |
| 2B | Pendleton 2017 | IWB57294 | RAC875_c37540_565 | 139.48 | 1.24E-06 | A/**G** | 0.11 | + | 7.56 |
| 2B | Pullman 2015 | IWB28962 | Excalibur_c80601_308 | 115.33 | 1.60E-08 | **T**/C | 0.42 | - | 5.91 |
| 2B | Pullman 2017 | IWB78516 | wsnp_Ex_c5856_10276064 | 126.53 | 2.69E-06 | **A**/G | 0.21 | + | 0.16 |
| 2D | Pullman 2016 | IWB44808 | Kukri_c38510_111 | 77.81 | 1.85E-06 | T/**C** | 0.46 | - | 6.63 |
| 3A | BLUP | IWB38639 | Ku_c18096_552 | 68.66 | 1.89E-06 | **T**/C | 0.45 | - | 1.45 |
| 4A | Pullman 2015 | IWB75368 | wsnp_BE405275A_Ta_1_1 | 29.86 | 3.71E-10 | **A**/C | 0.09 | - | 9.20 |
| 4A | Pullman 2017 | IWB78389 | wsnp_Ex_c539_1072859 | 60.39 | 2.13E-08 | **T**/C | 0.13 | + | 3.28 |
| 4A | Pullman 2017 | IWB60934 | RAC875_c88582_131 | 91.23 | 2.03E-06 | T/**C** | 0.06 | + | 3.43 |
| 5A | Lind 2017 | IWB48151 | Kukri_c8835_112 | 36.58 | 2.03E-06 | T/**C** | 0.20 | + | 4.38 |
| 5B | Pullman 2015 | IWB27067 | Excalibur_c49597_579 | 68.36 | 7.95E-10 | T/**C** | 0.05 | - | 8.53 |
| 5D | Pullman 2016 | IWB76253 | wsnp_Ex_c11055_17927668 | 204.58 | 4.64E-08 | **A**/G | 0.31 | - | 3.75 |
| 6A | BLUP | IWB51205 | Ra_c16143_1157 | 85.07 | 2.79E-08 | A/**G** | 0.32 | + | 7.29 |
| 6A | BLUP | IWB47595 | Kukri_c7458_1132 | 41.38 | 1.24E-07 | **T**/C | 0.43 | + | 3.17 |
| 6A | Pullman 2015 | IWB77245 | wsnp_Ex_c2236_4189774 | 126.17 | 2.06E-07 | T/**C** | 0.29 | + | 7.68 |
| 6A | Pullman 2016 | IWB72039 | Tdurum_contig50062_934 | 43.10 | 3.03E-07 | **T**/C | 0.38 | - | 3.90 |
| 6B | BLUP | IWB7778 | BS00030457_51 | 25.82 | 7.14E-07 | T/**C** | 0.26 | + | 5.17 |
| 6B | Pendleton 2017 | IWB57839 | RAC875_c42372_738 | 42.75 | 6.59E-08 | T/**G** | 0.14 | - | 4.42 |
| 6B | Pendleton 2017 | IWB77782 | wsnp_Ex_c33113_41653134 | 59.16 | 3.16E-07 | **T**/C | 0.44 | + | 0.72 |
| 7A | Lind 2017 | IWB44935 | Kukri_c39614_977 | 191.20 | 5.54E-07 | **T**/C | 0.34 | + | 0.94 |
| 7A | Pullman 2016 | IWB10213 | BS00068033_51 | 212.66 | 3.49E-09 | T/**G** | 0.45 | - | 4.94 |
| 7A | Pullman 2017 | IWB7243 | BS00022757_51 | 127.97 | 7.72E-08 | A/**G** | 0.10 | - | 4.32 |
| 7A | Pullman 2017 | IWB79640 | wsnp_JD_c12343_12604782 | 135.54 | 9.01E-08 | **T**/C | 0.16 | + | 3.48 |
| 7B | BLUP | IWB12006 | BS00097526_51 | 171.11 | 1.37E-07 | A/**G** | 0.17 | - | 1.01 |
| 7B | Lind 2017 | IWB78862 | wsnp_Ex_c8400_14157060 | 134.06 | 1.08E-07 | T/**C** | 0.29 | - | 2.18 |

^a,c,d,e^ Chromosome, SNP ID, SNP name, and chromosome position are all based on the wheat 90K consensus map [41]

^b^ Environment from which marker was identified as significantly associated with grain yield

^f^ Nominal p-values

^g^ Alleles for specific SNP markers; the underlined base represents the minor allele: the bold base represents the favorable allele if high yield is desired

^h^ Minor allele frequency (MAF). Frequency of minor allele in the panel

^i^ Alpha (α) denotes the SNP effect on the phenotype

^j^ Percent phenotypic variation explained by the SNP
