# Supplementary material for: Evaluating the Utility of Carbon Isotope Discrimination for Wheat Breeding in the Pacific Northwest
Source: Plant Phenomics. 2019 Aug 29;2019:4528719. doi: 10.34133/2019/4528719 (PMC7706333; doi:10.34133/2019/4528719)
Supplement: Supplementary 7 — Figure S2: quantile-quantile plots for association analysis of grain yield including no covariates for each location-year including (A) Pullman 2015, (B) Pullman 2016, (C) Pullman 2017, (D) Lind 2017, (E) Pendleton 2017, and (F) best linear unbiased prediction (BLUP). [file 4528719.f7.docx]

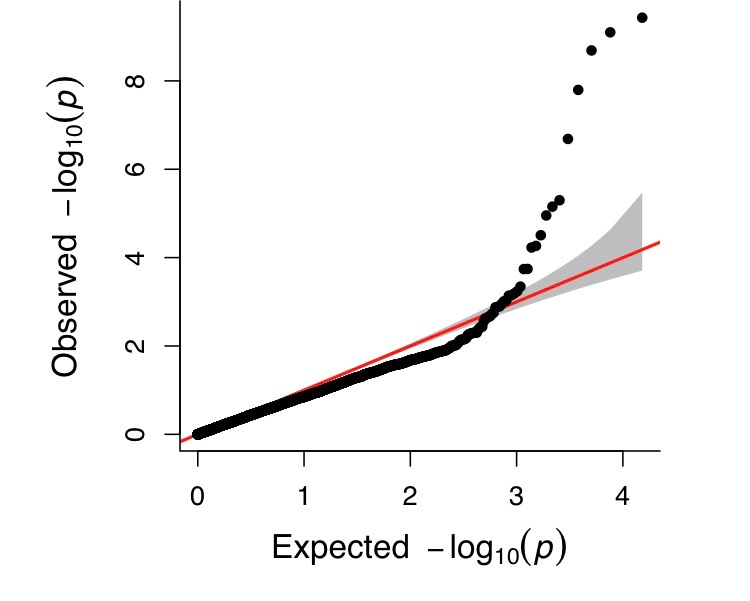


(**A**) Pullman 2015


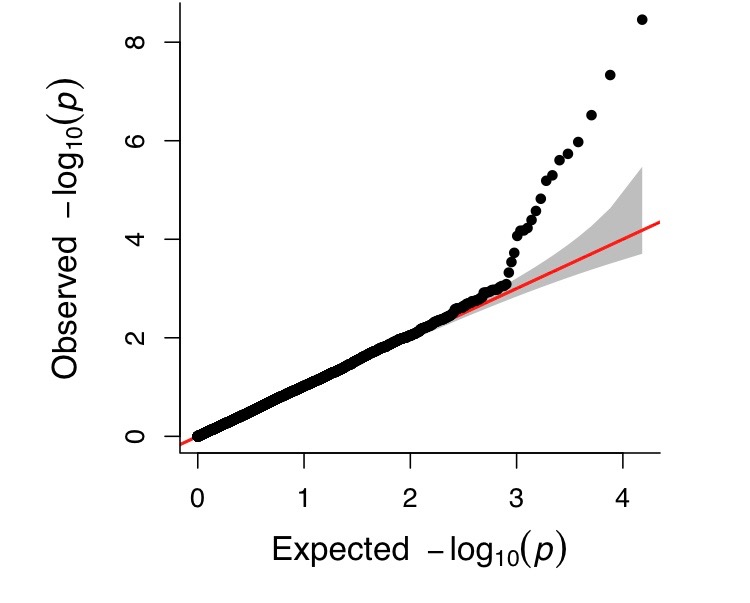


(**B**) Pullman 2016


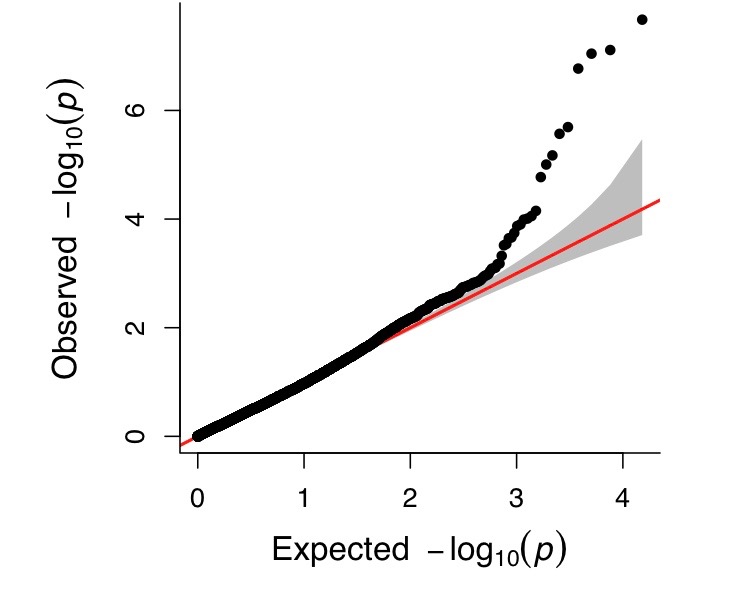


(**C**) Pullman 2017


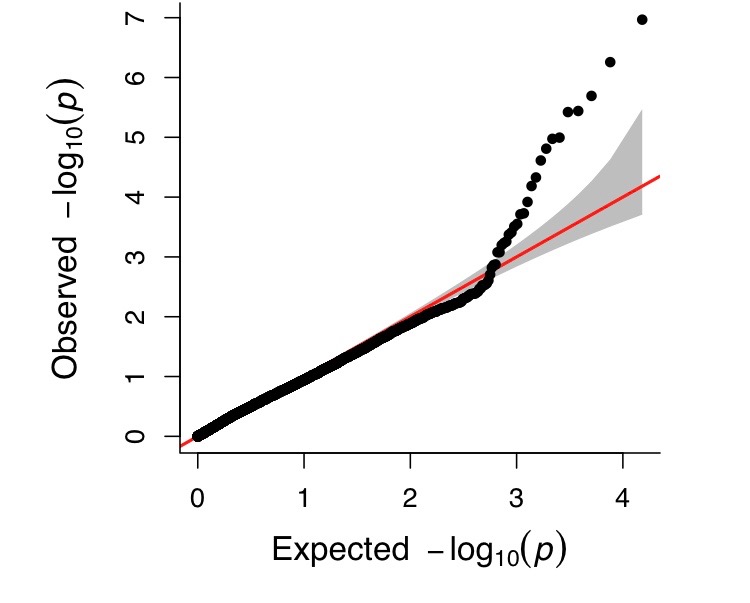


(**D**) Lind 2017


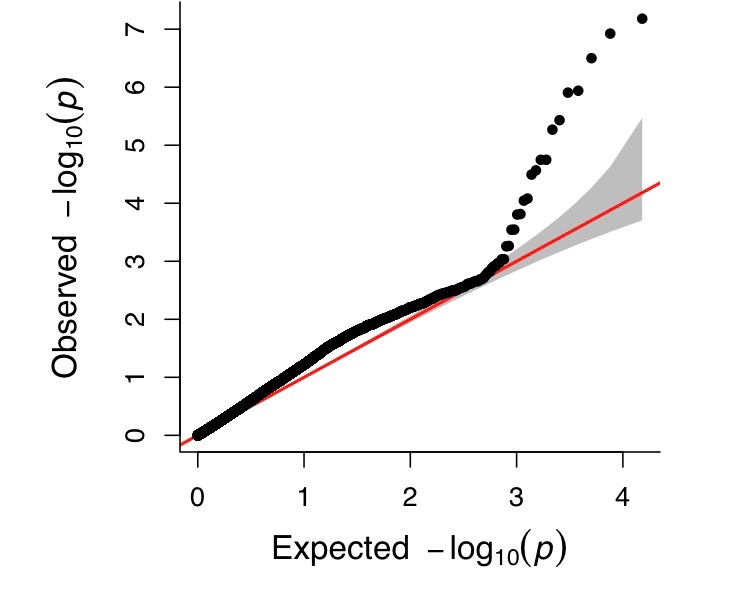


(**E**) Pendleton 2017


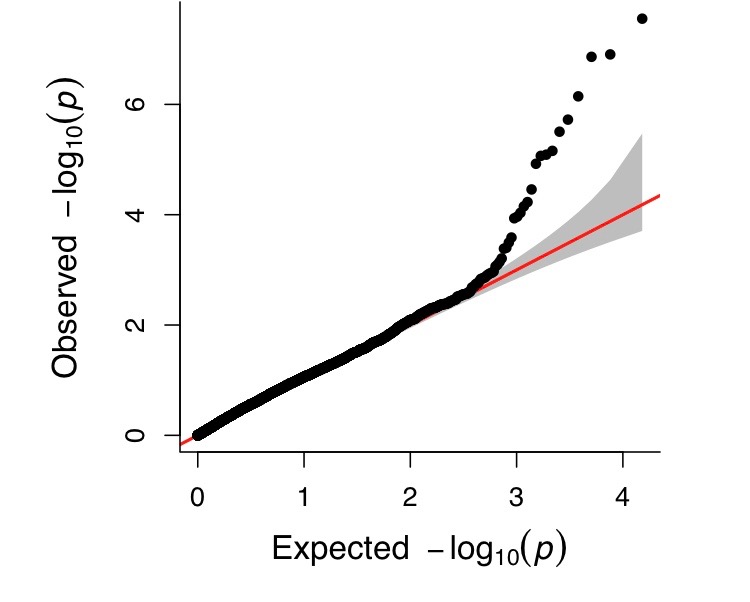


(**F**) BLUP

**Figure S2** Quantile-Quantile plots for association analysis of grain yield including no covariates for each location-year including (A) Pullman 2015 (B) Pullman 2016 (C) Pullman 2017 (D) Lind 2017, (E) Pendleton 2017, and (F) Best Linear Unbiased Prediction (BLUP)
